# Value of peripheral blood circulating tumor cell detection in the diagnosis of thoracic diseases and the prediction of severity

**DOI:** 10.1007/s10238-023-01022-3

**Published:** 2023-03-16

**Authors:** Chang Qi, Xian-Zhi Xiong

**Affiliations:** Department of Respiratory and Critical Care Medicine, NHC Key Laboratory of Pulmonary Diseases, Union Hospital, Tongji Medical College, Huazhong University of Science and Technology, 1277 Jiefang Avenue, Wuhan, 430022 Hubei China

**Keywords:** Circulating tumor cells, Thoracic disease, Metastases, Liquid biopsy, Biomarker

## Abstract

Circulating tumor cell (CTC) detection, as a noninvasive liquid biopsy method, has been used in the diagnosis, prognostic indication, and monitoring of a variety of cancers. In this study, we aimed to investigate whether CTC detection could be used in the early diagnosis and prediction of severity of thoracic diseases. We enrolled 168 thoracic disease patients, all of whom underwent pathological biopsy. Carcinoembryonic antigen (CEA) and neuron-specific enolase (NSE) measurement was also performed in 146 patients. There were 131 cases of malignant thoracic diseases and 37 cases of benign lesions. We detected CTCs in a 5 ml peripheral blood sample with the CTCBiopsy® system and analyzed the value of CTC count for predicting disease severity. Of 131 patients with a diagnosis of thoracic malignancy, CTCs were found in blood samples from 122 patients. However, only 2 out of 37 patients with benign thoracic disease had no detectable CTCs. There was no significant correlation between CTC count and benign and malignant lesions (*P* = 0.986). However, among 131 patients who had been diagnosed with malignant lesions, 33 had lymph node metastasis or distant metastasis. The presence of CTCs was significantly correlated with metastasis (*P* = 0.016 OR = 1.14). The area under the receiver operating characteristic (ROC) curve was 0.625 (95% confidence interval (CI), 0.519 to 0.730 *P* = 0.032). In addition, with stage IA1 as the cutoff, all patients were further divided into an early-stage group and a late-stage group. CTC count was significantly correlated with disease progression (*P* = 0.031 OR = 1.11), with an area under the curve (AUC) of 0.599 (95% CI, 0.506–0.692 *P* = 0.47). The sensitivity and specificity of CTC detection for the diagnosis of disease stage were 72.3% and 45.5%, respectively. In addition, the cutoff of 2.5 CTCs was the same when predicting disease metastasis and staging. Furthermore, the combination of CTC count, demographic characteristics and tumor markers had better predictive significance for disease staging. CTC count can effectively indicate the stages and metastasis of thoracic diseases, but it cannot differentiate benign and malignant diseases.

## Introduction

Thoracic diseases are common and frequently occurring diseases in humans. Lung cancer, in particular, is the malignancy with the highest morbidity and mortality worldwide [1]. Most clinically diagnosed cases of thoracic disease are in the advanced stage, and local recurrence and distant metastasis are the main causes of high mortality. Therefore, early diagnosis and early treatment are important strategies to effectively improve the prognosis of patients with thoracic malignant tumors. The diagnosis of thoracic disease usually relies on imaging, such as *computer tomography (*CT) and X-ray, mainly through the identification of lesions with abnormal shape and texture. The inconsistent visual characteristics of various lesions also make diagnosis a very challenging task. During the past decade, the use of low-dose computed tomography (LDCT) has promoted the development of lung cancer screening. The published results of the National Lung Screening Trial (NLST) demonstrated that LDCT screening for lung cancer, compared with X-ray, reduced the mortality rate of high-risk groups by 20% [2]. International academic organizations and many medical institutions have recommended LDCT screening for high-risk populations and developed the corresponding guidelines for lung cancer screening [3–6]. Although LDCT is effective in the diagnosis of thoracic diseases, there is still the problem of radiation exposure, which is not conducive to multiple follow-ups in a short time. In addition, due to the diversity of small nodules in imaging, LDCT is often associated with false-positive results on thoracic imaging [7], leading to overdiagnosis. With multislice spiral CT scanning technology, the outlines of lung tissue and lesions are clearly displayed. However, the missed diagnosis rate is relatively high when multislice spiral CT scanning is used for early screening of central lung cancer [8]. With the progress in the screening of groups with a high risk of thoracic disease, benign nodules have been detected in a large number of patients. Inappropriate diagnosis often leads to overdiagnosis or delayed treatment, which increases the anxiety of patients as well as the societal and economic burden. Therefore, the differential diagnosis of thoracic diseases has become a new opportunity and challenge for clinicians. It is necessary to identify the cancer high-risk population, to explore reasonable screening programs, and to reduce the economic burden.

With the development of liquid biopsy technology, the value of the application of various biomarkers has gradually been discovered by researchers. Biomarkers mainly include circulating tumor cells (CTCs), circulating tumor DNA (ctDNA), exosomes, miRNA, cell-free DNA (cfDNA) autoantibodies and so on [9]. As a major type of liquid biopsy, CTC detection can provide multilevel molecular information, including information on DNA, RNA, and protein, which is widely used for lung cancer, prostate cancer, ovarian cancer, and other cancers.

In 1869, Ashworth et al. first proposed the concept of circulating tumor cells. CTCs are tumor cells that are shed from primary tumors and enter the circulation, and from the time of circulation entry to tissue infiltration, a series of metastatic cascade processes will occur that result in distant metastasis and colonization. Tumors larger than 2 mm can induce angiogenesis, providing a basis for tumor cells to enter the circulation. Eyles et al. indicated that the migration of CTCs into the blood flow is an early event of human carcinogenesis [10]. A prospective study of 168 patients with chronic obstructive pulmonary disease (COPD) suggested that CTC detection can detect malignant chest lesions earlier than LDCT [11]. Several studies have reported that CTC detection can be used to monitor tumor heterogeneity and gene mutation [12], evaluate the response to chemotherapy and drug resistance, and predict treatment efficacy and cancer recurrence [13–15]. With the continuous progress in detection technology, the sensitivity and specificity of CTC detection have also been improved [16–18]. In fact, the seventh edition of the American Joint Committee on Cancer (AJCC) guidelines has included CTCs in the TNM staging system, proposing cM0 (I +) staging. In recent years, CTC detection has been included in the treatment guidelines and expert consensuses for a variety of cancers, such as esophageal cancer, breast cancer, and prostate cancer, as a prognostic predictor or an indicator for evaluating the efficacy of radiotherapy and chemotherapy [19–21].

We hypothesized that CTC detection alone or combined with tumor marker measurement could be used with imaging methods to improve the accuracy of thoracic disease differential diagnosis. In this study, we aimed to preliminarily determine the predictive value of CTC detection in the nature and staging of thoracic diseases.

## Materials and methods

### Patients and specimens

From February 2021 to November 2021, a total of 260 patients suspected of having thoracic diseases or with suspicious masses on CT were recruited from Union Hospital, Tongji Medical College, Huazhong University of Science and Technology. TNM staging of lung cancer patients was defined according to the Union for International Cancer Control (UICC) 2020 guidelines upon chest radiography, bronchoscopy, brain and thoracic computed tomography (CT), positron emission tomography CT (PETCT), and bone scintigraphy [1]. The study was approved by the Ethics Review Board (ERC) of Union Hospital, Tongji Medical College, Huazhong University of Science and Technology (No.2022–0716). A waiver for the requirement for informed consent was granted for this study due to the retrospective nature of the study, and any personal information within the data was deleted beforehand. The study conformed to the guidelines of the Declaration of Helsinki (as revised in 2013).

**Fig. 1:**
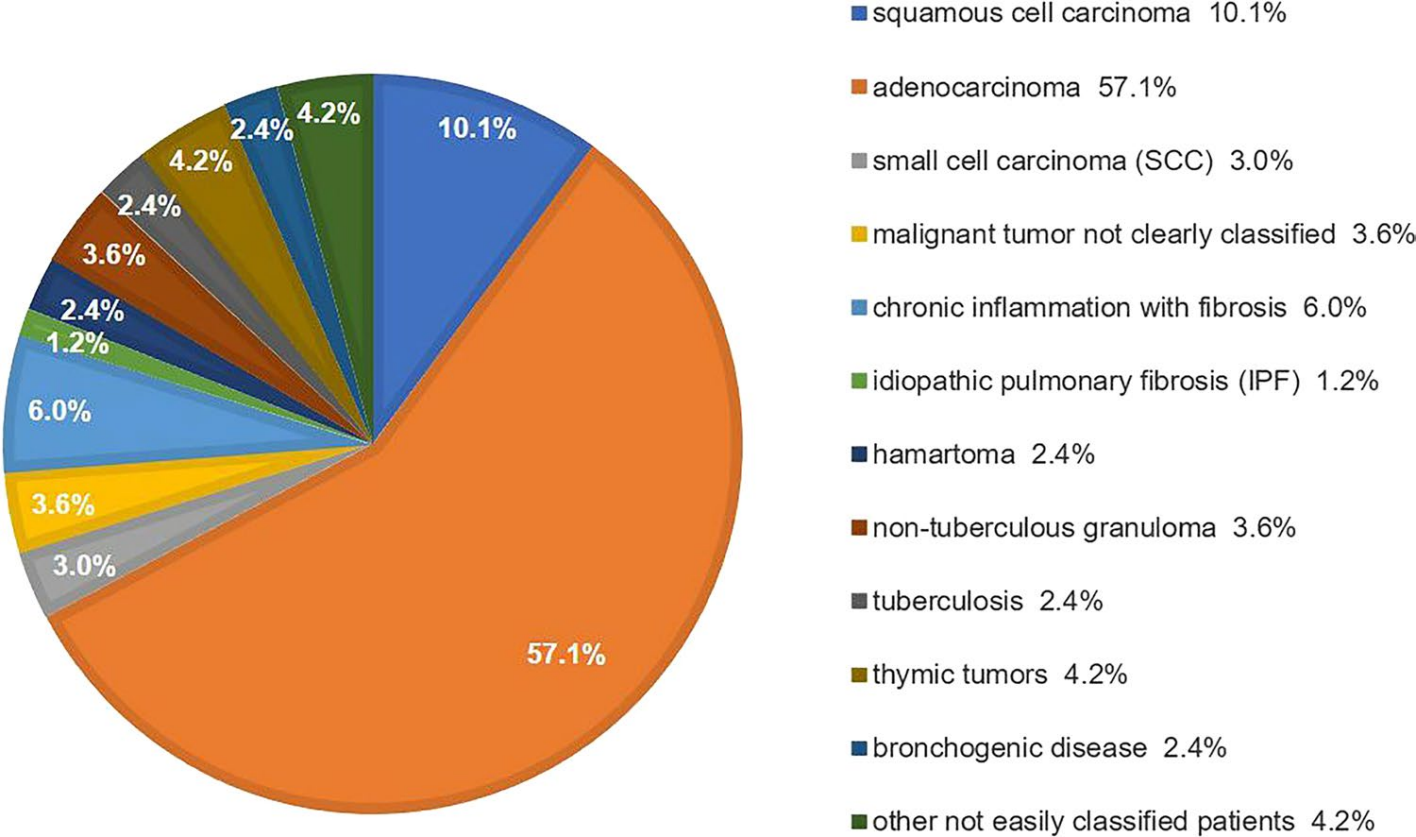
Diagnosis of patients undergoing pathological biopsy

Peripheral blood CTCs were detected in all patients at the first diagnosis, and subsequently, biopsy specimens were obtained by percutaneous needle biopsy under CT-guidance, pulmonary segmentectomy, bronchoscope, and endobronchial ultrasonography to complete the pathological diagnosis. We excluded patients with incomplete clinical data and long intervals between CTC detection and pathological diagnosis (> 3 months) and patients who had received antitumor therapy before CTC testing. Finally, 168 eligible patients were enrolled. There were 131 cases of malignant thoracic diseases and 37 cases of benign lesions. The cases of malignant thoracic disease included 37 carcinomas in situ, 53 stage I, 6 stage II malignant tumors, 25 stage III malignant tumors, and 10 stage IV malignant tumors. In all patients, pathological types included squamous cell carcinoma (*n* = 17), adenocarcinoma (*n* = 96), small cell carcinoma (SCC) (*n* = 5), malignant tumor not clearly classified (*n* = 6), idiopathic pulmonary fibrosis (IPF) (*n* = 2), chronic inflammation with fibrosis (*n* = 10), hamartoma (*n* = 4), tuberculosis (*n* = 4), nontuberculous granuloma (*n* = 6), thymic tumors (*n* = 7), bronchogenic disease (*n* = 4), and other not easily classified cases (such as sarcomatoid carcinoma, schwannoma, lymphoma, reactive lymphoid hyperplasia, and other diseases), as shown in Fig. 1. We defined "metastatic disease" as the presence of nodal involvement with cancer or the presence of distant metastases. Of the 131 patients with malignant thoracic disease in this study, 33 developed cancer metastases. In addition to CTC detection, carcinoembryonic antigen (CEA) and neuron-specific enolase (NSE) measurement was also performed for 146 patients.

### Collection and identification of CTCs

Before treatment for thoracic disease by any strategy, 5 mL of peripheral blood was collected from each patient and stored in EDTA anticoagulant tubes. To avoid sample degradation, sample processing and preparation were performed within 2 h of collection when stored at room temperature or within 24 h of collection when refrigerated at 4 °C.

Blood was filtered by the CTCBiopsy® system (Wuhan YZY Medical Technology Co., Ltd.). The system is based on the isolation by size of epithelial tumor cells (ISET) filtration method combined with the vacuum negative pressure filtration principle. By pressurizing the sample to pass through the filter membrane with a diameter of 8 μm, large-diameter cells such as CTCs, white blood cells and granulocytes are trapped in the blood, and red blood cells can be directly filtered out. Cells that met the following four cell morphological criteria were identified as CTCs and screened out: nucleus size ≥ 16 μm, anisonucleosis (ratio > 0.5),nuclear/cytoplasmic ratio > 2:1, nuclear heterogeneity, and the presence of three-dimensional sheets [22, 23]. A positive CTC result was defined as detection. Circulating tumor cell microemboli (CTM) are structures formed by the adhesion of multiple CTCs to cell‒cell adhesion proteins, including proteins involved in tight junctions and desmosomes [24, 25]. In this study, CTM were defined as clusters formed by > 3 CTCs.

### Measurement of serum tumor marker levels

Serum tumor marker measurements were performed at the same time as CTC detection. Serum was separated from 2 ml of clotted blood samples by centrifugation for 10 min at room temperature; then, serum tumor marker proteins, including NSE and CEA, for thoracic disease screening were measured using an automated clinical immunochemical analyzer. Positive diagnostic criteria for serum tumor markers were defined as levels that exceeded the normal reference range.

### Statistical analysis

SPSS 26.0 software was used for statistical analysis. All charts were drawn using GraphPad Prism and Microsoft Office. *P* < 0.05 was considered statistically significant. Receiver operating characteristic (ROC) curve and regression analyses were used to evaluate the correlation between CTC count and thoracic disease stage and the predictive value of CTC detection combined with tumor marker measurement for the final diagnosis of thoracic disease. Continuous variables were tested with the Shapiro–Wilk test. Continuous variable data with a normal distribution are presented as the mean ± standard deviation, and an independent sample *t* test was used to compare two groups. Data with a non-normal distribution are presented as the median and the upper and lower quartiles, namely, M (P25, P75), and the Mann‒Whitney *U* test was used for the comparison of groups.

## Results

### Correlation between CTCs and the nature of thoracic disease and metastasis

In the analysis of CTC detection results and pathological diagnosis, of 131 patients with a final diagnosis of thoracic malignancy, CTCs were found in blood samples from 122 patients. However, only 2 out of 37 patients with benign thoracic diseases had no detectable CTCs. For the detection of malignant tumors overall, the sensitivity of CTC detection was 93.13%, the specificity was 5.4%, the positive predictive value (PPV) was 77.7%, and the negative predictive value (NPV) was 18.2%. The results of the binary logistic regression analysis of CTC count and pathological results for 168 patients showed that there was no significant correlation between CTC count and benign or malignant lesions (*P* = 0.986), with an area under the curve (AUC) of 0.480 (95% confidence interval (CI), 0.377–0.582 *P* = 0.708).

**Fig. 2:**
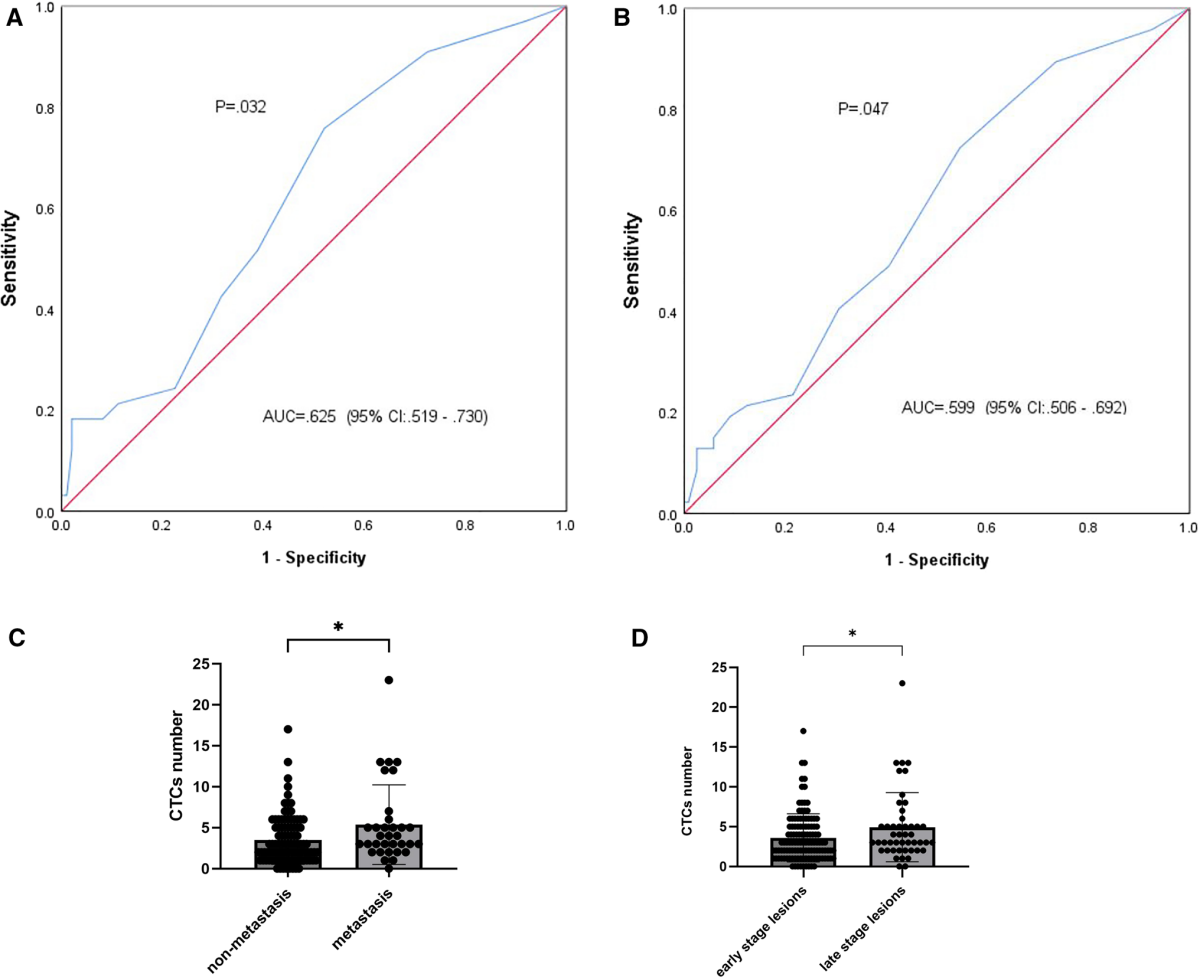
CTC detection in patients with thoracic disease. **A** ROC curve of predicting malignant thoracic disease metastasis by CTCs. **B** ROC curve of predicting thoracic disease staging by CTCs. **C** Differences in CTCs between patients with and without tumor metastasis. **D** Differences in CTCs between patients with early and late stage diseases. The grouping was bounded by clinical stage IA1. *AUC*, area under the curve; *95% CI*, 95% confidence interval; *CTCs*, circulating tumor cells; * *p*<0.05

Among 131 patients who had been diagnosed with malignant lesions, 33 had lymph node metastasis or distant metastasis. The results of the binary logistic regression analysis of CTC count and its relationship with malignant tumor metastasis confirmed that CTCs were significantly correlated with metastasis (*P* = 0.017 OR = 1.14). ROC curve analysis of CTC count was performed to evaluate the prediction of metastasis. When the number of CTCs detected was 2.5, the Youden index was the maximum, with an AUC of 0.625 (95% CI, 0.519 to 0.730 *P* = 0.032). At this number of CTCs, the sensitivity of CTCs for metastasis prediction was 75.0%, and the specificity was 47.5% (Fig. 2A). The number of CTCs in patients with malignant tumors increased with tumor metastasis (Fig. 2C and Table 1).

**Table 1:** Number of CTCs in patients with thoracic diseases

Groups	*N*	CTCs	*P* value	*Z*
Tumor stage				
Early-stage	121	3 (1,5)	0.045	− 2.004
Late-stage	47	3 (2,5)		
Tumor metastases				
Non-metastasis	33	3 (1,5)	0.031	− 2.162
Metastasis	98	4 (2.5,5.5)		
Histology				
Benign lesion	37	3 (2,5)	0.705	− 0.378
Malignant lesion	131	3 (2,5)		

*CTC*s: circulating tumor cells. Data of each group did not conform to the normal distribution and were expressed as median and upper and lower quartiles, such as M(P25, P75). The difference was statistically significant at *P* < 0.05

Moreover, CTM were detected in the peripheral blood of 5 patients, and all of these patients had pathological biopsy results showing thoracic malignant tumors.

### Correlation between CTCs and disease stage

We regrouped the cases according to TNM staging results. There were 37 patients with benign lesions, 37 patients with carcinomas in situ and microinvasive carcinomas, 47 patients with IA1 malignant tumors, 6 patients with stage I malignant tumors (non-IA1), 6 patients with stage II malignant tumors, 25 patients with stage III malignant tumors, and 10 patients with stage IV malignant tumors. The results of the multivariate linear analysis of the relationship between CTCs and thoracic disease stage showed that CTC count was significantly correlated with disease stage (*P* = 0.016 *R*-square = 0.034).

**Fig. 3:**
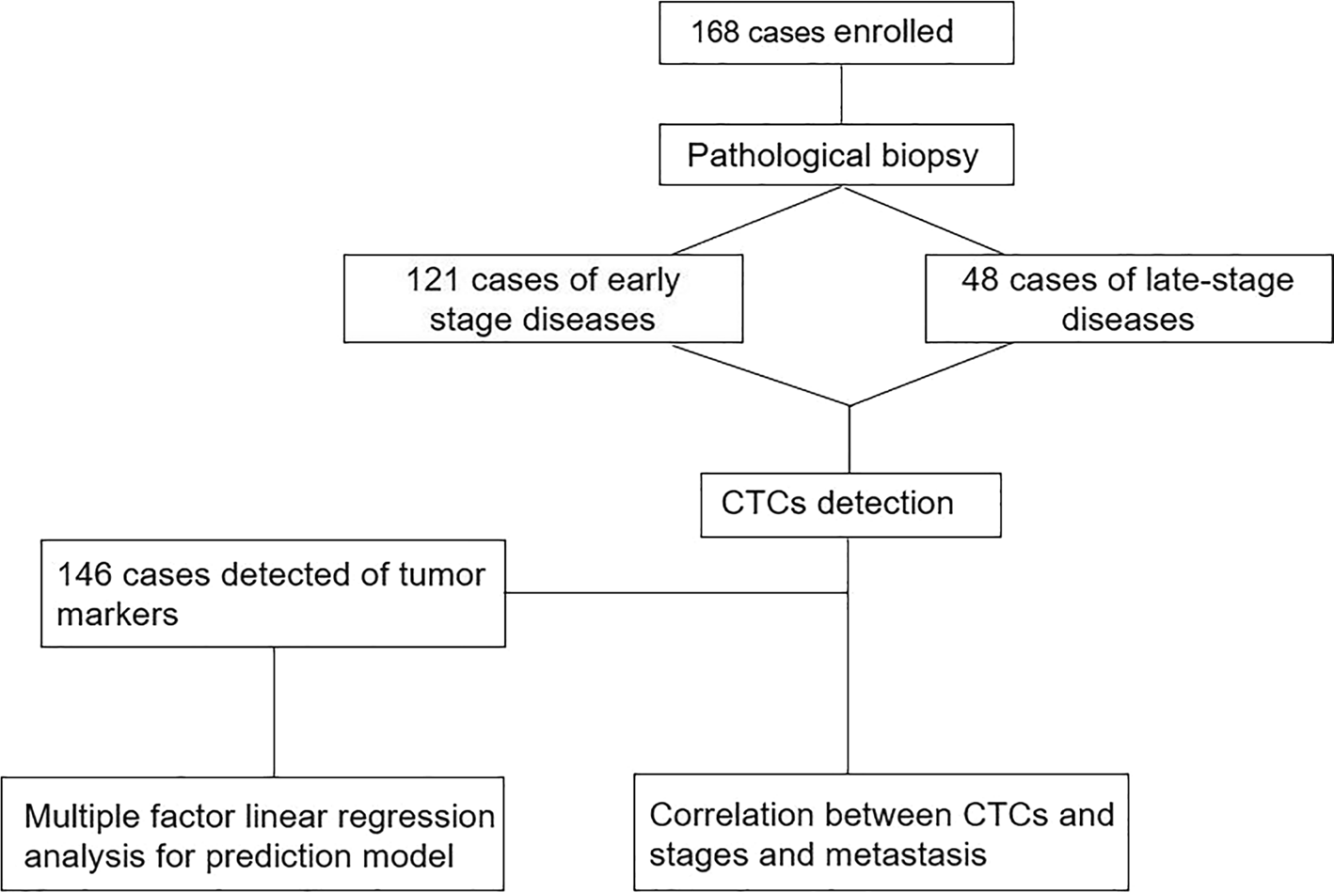
Experimental flowchart

With clinical stage IA1 as the cutoff, all patients were further divided into an early-stage group (including benign lesions, carcinoma in situ, and stage IA1 malignancies, *n* = 121) and a late-stage group (including stage I, II, III, and IV malignancies in addition to stage IA1, *n* = 48) (Fig. 3). The results of the binary logistic regression analysis of CTC count and tumor stage confirmed that CTC count was significantly correlated with disease progression (*P* = 0.031 OR = 1.11), with an AUC of 0.599 (95% CI, 0.506 to 0.692 *P* = 0.047) (Fig. 2B). CTC count in thoracic disease patients increased with disease progression (Fig. 2D and Table 4). According to the maximum Youden’s index, the cutoff value of 2.5 CTCs/5 ml blood yielded a sensitivity of 72.3% and specificity of 45.5%.

In addition, as shown in Table 2, the correlation analysis of multiple factors showed that the demographic data (age, sex) and serum tumor markers (CEA, NSE) of the enrolled patients were associated with the disease stage. The results of a multiple linear regression analysis showed that age, CEA, and NSE were significantly associated with disease stage, whereas sex was not, after excluding confounding factors. NSE was the independent variable with the greatest impact on the outcome. These findings suggested that CTCs combined with demographic characteristics and tumor markers have a better predictive significance for disease staging (*P* = 0.026 *R*-square = 0.259) (Table 3).

**Table 2:** Correlation between factors and stage of thoracic diseases

	CTCs	Age (years)	Gender	CEA	NSE
Pearson Correlation	.185	.263	−.272	.263	.330
*P* value	.016	.001	.000	.002	.000
*N*	168	167	168	143	147

*CTC*s, circulating tumor cells; *CEA*, carcinoembryonic antigen; *NSE*, neuronspecific enolase. The difference was statistically significant at *P* < 0.05

**Table 3:** Multiple linear regression models for thoracic diseases outcomes

Factors	Number	Beta	*T*	*P* value
CTCs	3(2,5)	.172	2.250	.026
Age (years)	57(50,63)	.181	2.327	.021
Gender	–	−.081	− 1.021	.309
CEA (µg/L)	1.9(1.3,2.9)	.223	2.960	.004
NSE (µg/L)	14.32(12.27,17.74)	.296	3.909	.000

*CTC*s, circulating tumor cells; *CEA*, carcinoembryonic antigen; *NSE*, neuronspecific enolase. Data of each group did not conform to the normal distribution and were expressed as median and upper and lower quartiles, such as M(P25, P75). The difference was statistically significant at *P* < 0.05

## Discussion

In this study, we used the CTCBiopsy® system to determine CTC count and investigated the ability of CTC count to predict pathological stage in patients with suspicious thoracic nodules or masses on CT. In particular, we evaluated the predictability of outcomes when CTC counts were combined with tumor markers. Pathological biopsy by percutaneous CT-guided lung puncture, lobectomy, and bronchoscopy is the gold standard for the diagnosis of nodules or masses suspected of being malignant. Notably, in all our patients, blood samples for the detection of CTCs were collected through routine venous access before or shortly after pathological biopsy was performed and before any treatment was administered to avoid the confounding effects of anti-infection, antitumor, and other therapeutic interventions.

The role of circulating tumor marker cell detection in the diagnosis of lung cancer has been reported previously. A total of 44 patients suspected of having lung cancer and 20 healthy volunteers were included in a study by Guo-Chen Duan et al [26]. The sensitivity and specificity of CTC detection in the diagnosis of early lung cancer were 52.94% and 90%, respectively. The AUC was 0.715 (95% CI 0.549–0.880, *P* = 0.041). The detection rate of CTCs increased with disease progression. Mario Mascalchi et al. showed that the sensitivity of CTC/CTM presence for malignancy was 70.1% (95% CI: 56.9–83.1%), the specificity was 100%, the positive predictive value was 100% and the negative predictive value was 28.6% (95% CI: 11.9–45.3%) [27]. However, when we considered all included patients, we obtained a sensitivity of 93.13% and a specificity of 5.4%. Compared with previous reports of early or advanced NSCLC, CTCs were detected at a higher rate in patients with thoracic disease, and the results of the binary logistic regression analysis of CTCs and outcomes were not significant in this study. This suggests that the presence of CTCs in peripheral blood may be a highly sensitive but nonspecific biomarker of biopsy-confirmed lung malignancy but not a valid predictor of the nature of thoracic disease.

We speculate that the low predictive value of CTCs detection in patients diagnosed with benign and malignant thoracic disease may be due to the following reasons. In recent years, due to the popularization of lung cancer screening, a large number of early thoracic diseases have been detected. Among the patients included in this study, patients with stage I tumors accounted for 40% of the total sample, which may partially explain the cutoff value of the disease stages. The CTC count in the peripheral blood of patients with early thoracic diseases was lower due to the limitation of their lesions; on the other hand, a few CTCs may also be detected in some patients with benign diseases due to inflammation, infection, detection error and other factors [28]. Therefore, the CTCBiopsy® system is not effective in distinguishing the nature of early lesions. Moreover, it is reasonable to speculate that these differences in results could also be related to the higher detection rate of CTCBiopsy® and the volume of the peripheral blood sample.

It has been shown that the CTC count increases significantly with increasing pathological stage. Therefore, it is reasonable to divide the enrolled patients into an early-stage group and a late-stage group according to the stage of disease progression to explore the correlation between CTC count and disease progression. We confirmed that the number of CTCs in peripheral blood was significantly associated with stage by linear regression analysis of data grouped with IA1 stage as the cutoff. In addition, unlike previous similar studies, we further confirmed that CTCs were significantly associated with disease metastasis. The results of the logistic regression model showed that the risk probability of tumor metastasis increased 1.14 times for every 1 unit increase in CTC count, which may be due to the higher tumor burden in patients with extensively metastatic tumors.

In 146 of 168 enrolled patients, both tumor markers and CTCs were tested. As shown in Table 3, the sensitivity of CTCs in the diagnosis of benign and malignant diseases was higher than that of CEA and NSE but with lower specificity. Multivariate analysis of NSE, CEA, CTCs, and disease stage showed that tumor markers and CTCs were significantly correlated with disease stage, and NSE had the greatest impact on the outcome after excluding confounding factors such as sex and age (*B* = 0.296). The predictive value of the multifactor linear model for staging outcome was 25.9%, which fitting the degree of the equation was significantly higher than that of the CTCs. Therefore, we believe that CTCs combined with tumor markers to judge the progression of thoracic disease will have greater clinical significance than either factor alone.

**Table 4:** The predictive effect of different test indexes in patients with thoracic diseases

*N* = 146	Sensitivity%	Specificity %	PPV%	NPV%
CTCs(5 ml Peripheral Blood)	93.9	6.5	78.8	22.2
CEA (µg/L)	9.6	90.3	78.6	21.2
NSE (µg/L)	34.8	67.7	80	21.9

*CTCs*, circulating tumor cells; *CEA*, carcinoembryonic antigen; *NSE*, neuronspecific enolase; *PPV*, positive predictive value; *NPV*, negative predictive value. Normal reference ranges for CEA: < 5 µg/L. Normal reference ranges for NSE: < 16.3 µg/L

CTM confers stemness properties to the cells. In particular, CD44, a cell surface marker often upregulated in cancer stem cells (CSCs), was overexpressed in CTM, which is involved in the maintenance of stemness signaling in numerous tumor cell types [29]. Similarly, stemness-associated genes were found to be upregulated in breast cancer PDX-derived and patient-isolated CTM [30]. The aggregation of CTCs may provide a good maintenance niche and provide necessary protection against distant tumor metastasis. Several studies have shown that, compared with individual CTCs, the CTCs clusters are rare in peripheral blood but more aggressive, more resistant to apoptosis, and more metastatic [31, 32]. In this study, CTM were detected in the peripheral blood of five patients. According to pathological biopsy, they were all patients with thoracic malignancy. Remarkably, we can speculate that the presence of CTM may be a more meaningful biomarker of thoracic tumors than the presence of CTCs. However, there is still a lack of stronger evidence-based medical evidence for this.

We recognize the following limitations of our study. Although CTCs contain DNA, RNA, protein, and other information, which can reflect tumors more comprehensively, they may be inferior to ctDNA in reflecting tumor heterogeneity. About tens of thousands of tumor cells are shed into the blood daily per gram of solid tumors. [33, 34]. However, CTCs are affected by immune surveillance, blood flow shear force, oxidative stress, and other aspects of blood circulation. So that it is not easy to accurately detect them in peripheral blood [35], which has been the bottleneck in the scientific exploration of CTCs and the main reason for their limited clinical function.There are false-positive results in the detection of CTCs. Although CTCs or CTM usually do not occur in healthy individuals, circulating nonhematologic cells (CNHCs), which have been reported in thyroid, parathyroid, and pancreatic diseases are sometimes misdiagnosed as CTCs [36, 37]. Frederick George Mayall et al. found that some metastatic cancer patients may have benign cytokeratin-positive cells in the circulation, which are also found in healthy volunteers [28]. Due to the limitations of detection technology, the CTCBiopsy® system may have a high detection rate, which is related to the inflammatory response produced by some benign diseases, tumor type, tumor size, tumor burden, the half-life of CTCs, and other factors. Other undetected tumors, multiple pulmonary nodules, and metastatic lung cancer can also cause interference. Of the 168 patients enrolled in our study, 96 (57.1%) patients had adenocarcinoma. Some studies have shown that the accuracy of CTC detection is best in patients with adenocarcinoma [38]. This selection bias may also lead to an overabundance of false-positives due to differences between cancer types. Clearly, addressing this issue requires the evaluation of data from a larger cohort of CTCs of patients with benign thoracic diseases.

In addition, compared with LDCT screening studies, most circulating tumor marker studies are small-population, single-center clinical studies. There is currently no high-quality evidence to support the implementation of the evaluation of these biomarkers in clinical practice. As a single screening and diagnosis method, CTC detection cannot meet the needs of early detection of lung cancer, and it is necessary to detect multiple biomarkers to achieve a prediction effect. As a result of the lack of valid data, our model of the prediction of thoracic disease stage is not perfect. We need a larger sample, more biomarkers, and a validation group to validate the model to increase the possibility of clinical application, which is what we intend to do in the future.

Overall, CTC count was not effective as a predictor of the histological nature of thoracic disease in this study, and CTC detection is expensive. Comprehensive assessment of CTCs and tumor markers may be beneficial. However, CTC count can indicate whether the disease is in early stages. In the clinical management of patients, the detection of multiple CTCs (> 2.5) in the peripheral blood of patients with imaging findings suggestive of thoracic space-occupying diseases can indicate a high probability of late-stage disease and metastasis. In contrast, the detection of no or few CTCs (< 2.5) in peripheral blood can indicate that the diagnosis is benign disease or early-stage malignant disease. In this case, the patient can be followed up for 1 to 3 months if they refuse to undergo pathological biopsy. If the disease progresses during follow-up, further management can be performed.

On the other hand, in patients who have been diagnosed with malignant thoracic disease, the detection of CTCs can suggest the presence of tumor metastasis, and subsequent surgical treatment can be considered. The detection of CTCs can be used as one of the measures to evaluate tumor systemic metastases and help clinicians accurately select the next treatment plan for patients in advance. Liquid biopsy is noninvasive and repeatable, which facilitates efficacy monitoring and follow-up [39]. The clinician should decide whether to perform CTC detection according to the patient's specific condition and main examination purpose.

## Conclusion

Unlike previous studies, our current study suggests that CTCs detected with the CTCBiopsy® system have the potential to be a biomarker for staging and metastasis prediction in patients with thoracic disease, mainly NSCLC, but have little relevance for the differentiation of benign and malignant nodules. Moreover, when the number of CTCs detected is 2.5, the maximum predictive value can be achieved. The combination of CTCs and tumor markers to judge the progression of thoracic diseases will have greater clinical significance than either biomarker alone.

## Data Availability

The data analyzed during the study are available from the corresponding author on reasonable request.
